# Annual Report of the Liverpool Institution for Diseases of the Eye, for July 1, 1821, to June 30th, 1822

**Published:** 1822-09

**Authors:** Alexander Hannay

**Affiliations:** Physician to the Institution


### STATISTICAL MEDICINE.

---

ART. I.—*Annual Report of the Liverpool Institution for Diseases of the Eye, for July* 1, 1821, *to June* 30*th*, 1822. By ALEXANDER HANNAY, M.D. Physician to the Institution.

ON comparing the present with our former report, (*vide Medical and Physical Journal*, vol. xlvii. p. 86,) you will perceive that the charity increases in utility; and I have pleasure in assuring you that it has every prospect of becoming more extensively beneficial, it being in contemplation to annex to it an infirmary, when there will be every necessary accommodation for those afflicted with the more severe forms of disease, as well as for those undergoing important and delicate operations, who, at present, are exposed to the influence of many causes highly unfavourable to their cure. We have had twelve operations for cataract and artificial pupil, all of which have succeeded remarkably well. The case of congenital cataract, the subject of which was only six months old, both of whose eyes were operated on, is one of the most perfect cures I ever witnessed.

I may take this opportunity of remarking, that we have been using the extract of stramonium pretty extensively in all those diseased states of the eye, in which the belladonna has usually been employed with such good effect, and have found it to be fully more active and efficient than that valuable medicine: indeed, in some cases it succeeded when the belladonna produced no alteration on the pupil. As, however, at some future period, I may recur to this subject, I shall not at the present trespass farther on your indulgence than by mentioning to you that I, a few months since, witnessed the most violent symptoms from the extract of stramonium taken internally, in the form of decoction, by mistake for the extract of sarsaparilla. The symptoms, however, happily yielded to the powerful remedies (most assiduously used,) usually had recourse to in counteracting the fatal effects of the narcotic poisons.

*Liverpool; 22d July,* 1822.

CASES.

| | | | |
|---|---|---|---|
| Acute Ophthalmia | 71 | Closure of the Pupil, with Obliteration of the anterior Chamber | 6 |
| Purulent Ophthalmia (Adults) | 54 | Staphyloma | 2 |
| Ditto (Infants) | 138 | Cataract | 28 |
| Mucous Ophthalmia | 6 | Ditto, Congenital | 2 |
| Ophthalmia, with Pustules | 49 | Glaucoma | 1 |
| Ditto, with Slough or Ulcer | 53 | Amaurosis, organic and functional | 52 |
| Ditto, with Hypopian | 7 | Paralysis of the upper Eyelids | 2 |
| Strumous Ophthalmia | 55 | Tinea | 50 |
| Chronic Ophthalmia | 65 | Lippitudo | 7 |
| Ditto, with granular Conjunctiva and vascular Cornea | 19 | Inversion of the Eyelids | 4 |
| Excrescence of the Conjunctiva | 1 | Eversion of the Eyelids | 2 |
| Perigium | 3 | Trechiasis | 3 |
| Opacities of the Cornea | 69 | Tumors in the Eyelids | 11 |
| Protusions of the Iris | 7 | Hordeolum | 6 |
| Inflammation of the Iris | 23 | Diseases of the Lacrymal Passages | 7 |
| Unnatural Adhesions of the Iris, with disfigured and contracted Pupil | 11 | Wounds and Injuries of the Eye | 13 |
| | | | 827 |
| Remaining upon the books on making up the last report | | | 150 |
| Total | | | 977 |

Of whom, 703 have been discharged cured,
72 more or less relieved,
34 incurable or irregular,
and 163 are now remaining upon the books.

Twelve cases were successfully operated on for the cataract and artificial pupil, including a patient born blind.

